# Relation of cardiac adipose tissue to coronary calcification and myocardial microvascular function in type 1 and type 2 diabetes

**DOI:** 10.1186/s12933-020-0995-x

**Published:** 2020-02-10

**Authors:** Emilie H. Zobel, Regitse Højgaard Christensen, Signe A. Winther, Philip Hasbak, Christian Stevns Hansen, Bernt J. von Scholten, Lene Holmvang, Andreas Kjaer, Peter Rossing, Tine W. Hansen

**Affiliations:** 1 Steno Diabetes Center Copenhagen, Niels Steensens Vej 2, 2820 Gentofte, Denmark; 2 The Centre of Inflammation and Metabolism and the Centre for Physical Activity Research, Rigshospitalet, Copenhagen, Denmark; 3 Department of Clinical Physiology, Nuclear Medicine & PET and Cluster for Molecular Imaging, Rigshospitalet and University of Copenhagen, Copenhagen, Denmark; 4 Department of Cardiology, Rigshospitalet, Copenhagen, Denmark; 5 University of Copenhagen, Copenhagen, Denmark

**Keywords:** Cardiac adipose tissue, Epicardial adipose tissue, Pericardial adipose tissue, Type 1 diabetes, Type 2 diabetes, Albuminuria, Myocardial flow reserve, Coronary artery calcium score, Cardiac sympathetic innervation, MIBG, Cardiac PET/CT

## Abstract

**Background:**

Cardiac adipose tissue may have local paracrine effects on epicardial arteries and the underlying myocardium, promoting calcification and affecting myocardial microcirculation. We explored whether the total amount of cardiac adipose tissue was associated with coronary artery calcium score (CAC) and myocardial flow reserve in persons with type 1 or type 2 diabetes and healthy controls.

**Methods:**

We studied three groups: (1) 30 controls, (2) 60 persons with type 1 diabetes and (3) 60 persons with type 2 diabetes. The three groups were matched for sex and age. The three groups derived from retrospective analysis of two clinical studies. All underwent cardiac ^82^Rb positron emission tomography/computed tomography (PET/CT) scanning. Cardiac adipose tissue volume (the sum of epicardial and pericardial fat), CAC, and myocardial flow reserve (ratio of pharmacological stress flow and rest flow) were evaluated using semiautomatic software. We applied linear regression to assess the association between cardiac adipose tissue, CAC and myocardial flow reserve.

**Results:**

Mean (SD) cardiac adipose tissue volume was 99 (61) mL in the control group, 106 (78) mL in the type 1 diabetes group and 228 (97) mL in the type 2 diabetes group. Cardiac adipose tissue was positively associated with body mass index in all three groups (p ≤ 0.02). In the controls, cardiac adipose tissue was positively associated with CAC score (p = 0.008) and negatively associated with myocardial flow reserve (p = 0.005). However, cardiac adipose tissue was not associated with CAC or myocardial flow reserve in the groups including persons with type 1 or type 2 diabetes (p ≥ 0.50).

**Conclusions:**

In contrast to what was found in healthy controls, we could not establish a relation between cardiac adipose tissue and coronary calcification or myocardial microvascular function in person with type 1 or type 2 diabetes. The role of cardiac adipose tissue in cardiovascular disease in diabetes remains unclear.

## Background

Cardiac adipose tissue is a highly metabolic active fat depot surrounding the heart and coronary arteries. It includes epi- and pericardial adipose tissue. There has been considerable interest in the cardiac adipose tissue as a risk marker [1]. High levels of epicardial adipose tissue have been associated with incident cardiovascular disease and mortality in persons (particularly men) with type 2 diabetes in our previous study [2] and was associated with increased risk of coronary artery disease in persons with a high risk of cardiovascular disease, of whom 45% had type 2 diabetes [3]. An increased epicardial adipose tissue volume has also been demonstrated to be an important determinant of atrial fibrillation recurrence following electrical cardioversion or catheter ablation [4] and to correlate with ventricular tachycardia recurrence after catheter ablation in persons undergoing ventricular tachycardia ablation [5]. In contrast, a study in persons with type 1 diabetes could not demonstrate an association between the epicardial adipose tissue volume and coronary atherosclerosis [6].

Little is known about the possible mechanisms that could link cardiac adipose tissue to an increased risk of cardiovascular disease. It has been demonstrated that adipose tissue is an active endocrine tissue, implied in the production of numerous pro-inflammatory mediators [7]. Thus, abdominal adipose tissue in persons with overweight has been linked to an over-production of inflammatory mediators with effects on the local fat, insulin resistance and there has been an increasing interest in a possible crosstalk with myocardial function [8, 9]. These effects may be reduced with hypoglycemic agents with pleiotropic effects.

Epicardial adipose tissue embeds the coronary arteries and autonomic nerve fibers, and shares microcirculation with the underlying myocardium. We were interested in whether the association between cardiac adipose tissue and cardiovascular disease might be mediated by micro- or macrovascular changes or autonomic nerve damage. Due to the proximity of the structures, cardiac adipose tissue could through paracrine signalling promote calcification or impair the myocardial microcirculatory function [7, 10]. Knowledge of these associations in persons with diabetes, who are known to have higher amounts of cardiac adipose tissue and higher risk of cardiovascular disease compared to persons without diabetes, is sparse [6, 11–16]. A study in patients with systolic heart failure demonstrated an association between epicardial adipose tissue thickness and cardiac sympathetic denervation, implying that cardiac adipose tissue could affect autonomic nerve function [17].

The aim of this retrospective analysis was to explore whether the total amount of cardiac adipose tissue (sum of epi- and pericardial adipose tissue) was associated with coronary artery calcium score (CAC), myocardial flow reserve and/or cardiac sympathetic nerve integrity in persons with type 1 or type 2 diabetes and healthy controls.

## Methods

### Study population

The present study is a retrospective analysis of two clinical studies performed at a single center: A study from 2013 in 60 persons with type 2 diabetes and 30 controls [18]; and a study from 2016 to 2018 in 60 persons with type 1 diabetes [19]. The two studies used identical protocols and the same equipment and were therefore suitable for comparisons. The clinical information was obtained at a visit at the Steno Diabetes Center Copenhagen and all scans were performed at the Department of Clinical Physiology, Nuclear Medicine & PET at Rigshospitalet, Copenhagen. In the present study, we analyzed all participants from the two original studies. Therefore, we had three groups to compare: (1) controls; (2) persons with type 1 diabetes; and (3) persons with type 2 diabetes. The three groups were matched for age and sex as part of the original design of the two studies.

Main exclusion criteria were renal failure and contraindications for cardiac positron emission tomography (PET)/computed tomography (CT). Cardiovascular disease [history of coronary artery disease or other cardiovascular disease (including stroke) or heart symptoms] was an exclusion criterion for the type 2 diabetes group and the controls, but not for the type 1 diabetes group. None of the participants had symptoms of angina at time of inclusion. History of cardiovascular disease was assessed from patient electronic health records and information on symptoms from the heart was obtained from thorough interviews with all participants using the WHO Rose questionnaire.

### Hybrid cardiac PET/CT imaging

Cardiac adipose tissue was measured using the cardiac software, Syngo.via Frontier—Cardiac risk assessment (Siemens, AG; Healthcare Sector, Germany), which uses non-contrast CT data to automatically quantify the adipose tissue volume located on the outside of the myocardium by tracing myocardial and adipose tissue borders based on the specific density of fat (− 150 to − 50 Hounsfield units). The software does not distinguish epi- and pericardial fat thus total cardiac adipose tissue above the entire heart was measured. Two separate investigators examined and verified the accuracy of the automatically generated adipose tissue tracings.

All participants underwent a dynamic, gated cardiac PET/CT on a hybrid PET/CT scanner in 3D mode (Siemens Biograph mCT 128, Siemens, Munich, Germany) after administration of 1100 MBq ^82^Rb (CardioGen-82, Bracco Diagnostics, Monroe Township, NJ, USA). Cardiac PET/CT was performed at rest and during stress (maximum myocardial hyperaemia was induced by adenosine infusion). Myocardial blood flow was calculated as the ratio between maximal induced and resting myocardial blood flow, as previously described in detail [18]. The myocardial flow reserve reflects to what extent the myocardial blood flow can increase during stress. Using the method described by Agatston, we calculated CAC score as the sum of CAC content in the three main coronary arteries using semiautomated software (Corridor4DM, INVIA, Ann Arbor, MI, USA), as previously reported [18]. Myocardial perfusion abnormalities were assessed semi quantitatively by two experienced operators.

### Cardiac sympathetic innervation by ^123^I-MIBG scintigraphy

We performed cardiac radionuclide imaging using the nonmetabolized norepinephrine analogue metaiodobenzylguanidine (MIBG). Sympathetic nerve terminals actively take up MIBG, and by quantifying the cardiac uptake, it is possible to directly assess cardiac sympathetic nerve integrity. MIBG is radioactively labeled with ^123^I to allow imaging. Cardiac ^123^I-MIBG uptake was semiquantified using images taken 240 min after intravenously tracer injection (200 MBq ^123^I-MIBG). We calculated the heart to mediastinum ratio, by drawing regions of interest following the epicardial contour and the upper mediastinum (avoiding the thyroid gland) in the planar anterior view [20]. An intact sympathetic innervation of the myocardium is reflected by a high heart-to-mediastinum ratio.

As previously described, cardiac ^123^I-MIBG scintigraphy was performed in all 60 participants with type 1 diabetes, in 29 of the participants with type 2 diabetes, and in 14 of the controls [20, 21].

### Clinical measurements

HbA_1c_ and lipids were measured by standard methods. The CKD-EPI equation was used to calculate eGFR. In the type 1 diabetes group, urine samples were taken three consecutive mornings and urinary albumin creatinine rate (UACR) was measured by an enzyme immunoassay. For the persons with type 2 diabetes and the controls, two 24-h urine collections were taken and urinary albumin excretion rate (UAER) was measured by an enzyme immunoassay. The 24-h blood pressure was recorded using a cuff-device (Takeda, TM2430, Japan) in the type 1 diabetes group and a tonometric wrist-device (BPro, HealthStats, Singapore) in the type 2 diabetes group and in the controls.

### Statistical analysis

Normal distributed continuous variables are provided as mean and standard deviation (SD), non-normal distributed (UACR, UAER and CAC) as median with interquartile range (IQR) and categorical variables as total numbers and percent. UACR, UAER and CAC were log2 transformed in all analyses.

Independent samples t-test was applied to compare continuous variables between participants with cardiac adipose tissue below or above the median, and the χ^2^ test or Fisher’s exact test, as appropriate, when comparing categorical variables.

Linear regression analysis was used to determine whether any association existed between total cardiac adipose tissue and clinical characteristics or measures of cardiac function. The three groups were analyzed separately.

A two-tailed p < 0.05 was interpreted as significant. Missing data was not replaced.

## Results

The characteristics of the controls, and the participants with type 1 and 2 diabetes are shown for all within the groups and according to median cardiac adipose tissue level in the three groups in Table 1. Persons with cardiac adipose tissue below the median had lower body mass index (BMI) in all three groups (p ≤ 0.02) and higher HDL-cholesterol level in the control group and in the type 2 diabetes group (p ≤ 0.04). The controls with cardiac adipose tissue below the median had lower CAC score and higher myocardial flow reserve (p = 0.005 for both).

**Table 1:** Clinical characteristics of all participants

Characteristics	Controls (n = 30)				Persons with type 1 diabetes (n = 60)				Persons with type 2 diabetes (n = 60)			
	All	Cardiac adipose tissue < 88 mL	Cardiac adipose tissue ≥ 88 mL	p	All	Cardiac adipose tissue < 71 mL	Cardiac adipose tissue ≥ 71 mL	p	All	Cardiac adipose tissue < 226 mL	Cardiac adipose tissue ≥ 226 mL	p
Numbers of participants	30	14	15	–	60	30	30	–	60	30	30	
Female, n (%)	12 (40.0)	7 (24.1)	4 (13.8)	0.20	25 (41.7)	16 (53.3)	9 (30.0)	0.07	20 (33.3)	13 (43.3)	7 (23.3)	0.1
Age (years)	60 (10)	57 (9)	63 (10)	0.14	59 (9)	58 (10)	61 (8)	0.21	63 (9)	64 (10)	63 (8)	0.76
Body mass index (kg/m^2^)	24.8 (3.4)	22.2 (2.0)	27.2 (2.8)	< *0.0001*	26.4 (4.2)	23.8 (2.6)	29.1 (3.8)	< *0.0001*	31.6 (4.5)	30.2 (4.5)	32.9 (4.2)	*0.02*
Known diabetes duration (years)	–	–	–	–	37 (14)	37 (13)	37 (14)	0.99	14 (10)	15 (8)	13 (12)	0.68
24 h systolic blood pressure (mmHg)	126 (14)	123 (11)	132 (14)	0.06	136 (10)	136 (10)	138 (10)	0.47	134 (18)	133 (16)	135 (20)	0.62
HbA_1c_ (%)	35.8 (1.9)	35.2 (2.2)	36.3 (1.5)	0.11	61.8 (10.1)	62.1 (9.7)	61.6 (10.6)	0.84	55.5 (11.4)	56.1 (13.3)	55.0 (9.3)	0.71
LDL cholesterol (mmol/L)	3.4 (0.7)	3.3 (0.5)	3.5 (0.9)	0.42	2.2 (0.8)	2.1 (0.6)	2.2 (0.9)	0.53	2.2 (0.9)	2.1 (0.8)	2.3 (0.9)	0.30
HDL cholesterol (mmol/L)	1.6 (0.5)	1.8 (0.7)	1.4 (0.3)	*0.04*	1.8 (0.5)	1.9 (0.5)	1.7 (0.5)	0.11	1.1 (0.4)	1.2 (0.5)	1.0 (0.2)	*0.03*
Total cholesterol (mmol/L)	5.5 (0.7)	5.5 (0.8)	5.5 (0.8)	0.96	4.5 (0.9)	4.4 (0.8)	4.5 (1.0)	0.47	4.3 (0.9)	4.2 (0.9)	4.4 (0.9)	0.60
eGFR (mL min^−1^ 1.73 m^−2^)	82.8 (13.1)	85.3 (12.2)	80.2 (14.3)	0.31	75.8 (22.3)	76.9 (24.0)	74.7 (20.7)	0.71	76.0 (24.0)	80.3 (23.0)	71.8 (24.6)	0.17
Urinary albumin excretion rate (mg/24 h)*	6.0 [5.0–10.5]	5.8 [5.0–6.5]	6.5 [5.0–22.5]	0.12	11.5 [3.3–120.8]	8.5 [4.3–129.7]	18 [3.0–106.0]	0.57	32.5 [6.5–146.0]	16 [6.5–93.5]	50.5 [8.5–208.3]	0.71
Anti-hypertensive treatment, n (%)	3 (10.0)	2 (6.9)	1 (3.4)	0.45	47 (78.3)	23 (76.7)	24 (80)	0.75	55 (91.7)	25 (83.3)	30 (100)	0.05
Aspirin treatment, n (%)	1 (3.3)	1 (3.5)	0 (0)	1.0	30 (50.0)	14 (46.7)	16 (53.3)	0.61	53 (88.3)	26 (86.7)	27 (90.0)	1.0
Lipid lowering medication, n (%)	0 (0)	0	0	–	45 (75.0)	22 (73.3)	23 (76.7)	0.77	56 (93.3)	28 (93.3)	28 (93.3)	1.0
Smokers, n (%)	4 (13.3)	3 (10.3)	1 (3.5)	0.33	8 (14.0)	4 (14.3)	4 (13.8)	1.0	46 (76.7)	23 (76.7)	23 (76.7)	1.0
Known coronary artery disease or ischemia identified by cardiac PET, n (%)	1 (3.3)	0	1	1.0	9 (15)	3 (10.0)	6 (20.0)	0.47	13 (21.7)	9 (30.0)	4 (13.3)	0.12
Left ventricle ejection fraction (mL)	60.0 (7.5)	59.8 (8.4)	60.2 (7.0)	0.9	66.1 (9.3)	68.2 (9.1)	64.0 (9.1)	0.08	61.6 (9.0)	60.2 (8.5)	63.0 (9.5)	0.25
Coronary artery calcium score	0 [0–81]	0 [0–0]	56 [0–176]	*0.005*	99 [22.5–434]	73 [17.5–392.5]	196.5 [23.5–1286.9]	0.63	211 [23.5–585]	300 [28–964]	163 [13–467]	0.35
Myocardial flow reserve	3.0 (0.8)	3.4 (0.7)	2.6 (0.7)	*0.005*	2.6 (1.0)	2.6 (1.1)	2.6 (0.9)	0.98	2.3 (0.7)	2.2 (0.6)	2.4 (0.8)	0.32
Late heart-to-mediastinum ratio n = 103	2.8 (0.6)	3.0 (0.5)	2.5 (0.7)	0.12	2.5 (0.5)	2.5 (0.5)	2.4 (0.4)	0.16	2.5 (0.5)	2.4 (0.6)	2.5 (0.5)	0.65

Data represents numbers, n (%), mean (SD) or median [IQR]

The three groups were analyzed separately, and p values compare values from participants with cardiac adipose tissue below or above the median within each of the three groups. p values from independent samples t-test and χ^2^ test or Fisher’s exact test

Italic values indicate significance of p value (p < 0.05)

*eGFR* estimated glomerular filtration rate

*Urinary albumin creatinine rate for the type 1 diabetes cohort

Reversible ischemia was observed on the cardiac PET/CT in 1 control person, 10 participants with type 1 diabetes (7 of whom were known with coronary artery disease) and 11 participants with type 2 diabetes. Five participants with type 1 diabetes and three with type 2 diabetes had irreversible ischemia (fixed perfusions defects) which was not found in any control persons.

Mean (SD) cardiac adipose tissue level was 99 (61) mL in the control group; 106 (78) mL in the persons with type 1 diabetes and 228 (97) mL in the persons with type 2 diabetes. The cardiac adipose tissue level was comparable in controls and persons with type 1 diabetes, however significantly higher in persons with type 2 diabetes compared to controls (p < 0.0001) and type 1 diabetes (p < 0.0001). These differences remained significant after adjustment for age, sex, BMI and UACR/UAER (p ≤ 0.02).

**Fig. 1:**
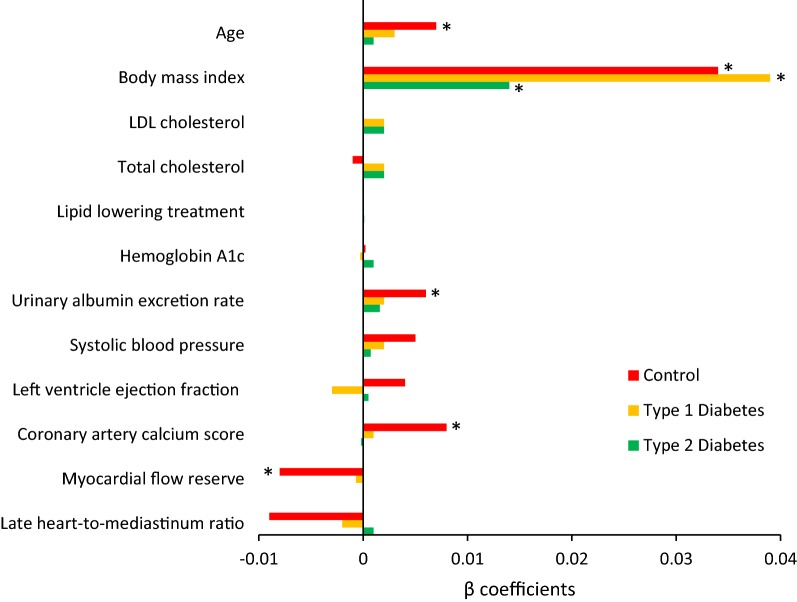
Associations between cardiac adipose tissue, clinical characteristics and measures of cardiac function. The three groups were analyzed separately using linear regression models and the β coefficients represent standardized effects within the groups. For lipid lowering treatment the β coefficients represent with versus without treatment. *p < 0.05

We evaluated the associations between cardiac adipose tissue and clinical characteristics separately in the three groups (Table 2 and Fig. 1). Cardiac adipose tissue was positively associated with BMI in all three groups (p ≤ 0.02). In the control group, cardiac adipose tissue was positively associated with age and UAER (p ≤ 0.03).

**Table 2:** Associations between cardiac adipose tissue and clinical characteristics as well as measures of cardiac function

	Controls (n = 30)		Persons with type 1 diabetes (n = 60)		Persons with type 2 diabetes (n = 60)	
	β coefficient (95% CI)	p	β coefficient (95% CI)	p	β coefficient (95% CI)	p
Clinical characteristics						
Age	0.007 (0.001; 0.013)	*0.02*	0.003 (− 0.0001; 0.006)	0.06	0.001 (− 0.001; 0.004)	0.27
Body mass index	0.034 (0.017; 0.052)	*0.0004*	0.039 (0.029; 0.049)	< *0.0001*	0.014 (0.003; 0.026)	*0.02*
LDL cholesterol	0.00006 (− 0.006; 0.007)	0.99	0.002 (− 0.001; 0.005)	0.20	0.002 (− 0.0008; 0.004)	0.17
Total cholesterol	− 0.001 (− 0.008; 0.005)	0.66	0.002 (− 0.001; 0.006)	0.19	0.002 (− 0.0008; 0.004)	0.16
Lipid lowering treatment	–	–	− 0.00005 (− 0.002; 0.001)	0.95	0.0001 (− 0.0005; 0.0008)	0.68
Hemoglobin A_1c_	0.0002 (− 0.004; 0.009)	0.46	− 0.0003 (− 0.004; 0.003)	0.84	0.001 (− 0.001; 0.003)	0.37
Urinary albumin excretion rate^a^	0.006 (0.0005; 0.012)	*0.03*	0.002 (− 0.002; 0.005)	0.31	0.0016 (− 0.001; 0.004)	0.38
Systolic blood pressure	0.005 (− 0.002; 0.011)	0.14	0.002 (− 0.002; 0.005)	0.29	0.0007 (− 0.002; 0.003)	0.60
Cardiac measures						
Left ventricle ejection fraction	− 0.004 (− 0.01; 0.003)	0.23	− 0.003 (− 0.006; 0.0003)	0.08	0.0005 (− 0.002; 0.003)	0.72
Coronary artery calcium score	0.008 (0.002; 0.013)	*0.008*	0.001 (− 0.002; 0.005)	0.50	− 0.0002 (− 0.003; 0.003)	0.89
Myocardial flow reserve	− 0.008 (− 0.014; − 0.003)	0.005	− 0.0007 (− 0.004; 0.003)	0.67	0.00002 (− 0.003; 0.003)	0.98
Late heart-to-mediastinum ratio^b^	− 0.009 (− 0.022; 0.003)	0.12	− 0.002 (− 0.006;0.001)	0.19	0.001 (− 0.003; 0.005)	0.59

Linear regression analysis. The three groups were analyzed separately. β coefficient represents standardized effects within groups

Italic values indicate significance of p value (p < 0.05)

^a^Urinary albumin creatinine rate for the persons with type 1 diabetes

^b^Available in 14 controls, 29 persons with type 2 diabetes and 60 persons with type 1 diabetes

Furthermore, we evaluated the associations between cardiac adipose tissue and myocardial flow reserve, left ventricular ejection fraction, CAC score and ^123^I-MIBG uptake, separately in the three groups (Table 2 and Fig. 1). Cardiac adipose tissue was positively associated with CAC score (p = 0.008) and negatively associated with myocardial flow reserve in the controls (p = 0.005). No significant associations were found for the persons with type 1 or type 2 diabetes. Cardiac adipose tissue was not associated with left ventricular ejection fraction or ^123^I-MIBG uptake in the three groups (p ≥ 0.08).

## Discussion

The main finding of our study was that total cardiac adipose tissue was not associated with advanced measures of cardiac micro- and macrovascular function including CAC score, myocardial flow reserve, and cardiac sympathetic integrity in persons with type 1 or type 2 diabetes. In contrast we found that cardiac adipose tissue was positively associated with CAC score and negatively associated with myocardial flow reserve in the controls.

### Cardiac adipose tissue and coronary atherosclerosis

The hypothesis that cardiac adipose tissue influences the development of coronary atherosclerosis through endocrine and paracrine activities has provoked considerable interest in the association between cardiac adipose tissue and coronary atherosclerosis. Large studies including the Framingham Heart Study have reported distinct associations between perivascular fat and CAC score in the general population [22]. We extended this association to apply for total cardiac adipose tissue in our control group. Evidence from persons with type 1 and type 2 diabetes is still sparse and conflicting, and mostly address epicardial and not total cardiac adipose tissue [13]. Our finding that cardiac adipose tissue and CAC score did not correlate in persons with type 1 diabetes confirms findings from a recent study using CT angiography to evaluate epicardial adipose tissue and CAC in 88 persons with type 1 diabetes [6]. Results from studies in type 2 diabetes and mixed populations (diabetes and non-diabetes) are conflicting. In the studies reporting an association between epicardial adipose tissue and coronary artery disease, including high risk plaque characteristics and CAC [13–15], it remains uncertain if the associations demonstrated are truly independent from traditional risk factors. We report a lack of association between cardiac adipose tissue and CAC score in persons with type 2 diabetes free of cardiovascular disease in line with results from a study in a mixed population (34% had diabetes) with suspected or known coronary artery disease (referred for clinically indicated invasive coronary angiography) [16].

### Cardiac adipose tissue and myocardial microcirculation

Due to the proximity of cardiac adipose tissue and the epicardial arteries and underlying myocardium, it is possible that cardiac adipose tissue has local effects on myocardial microcirculation and thereby influences the development of myocardial ischemia. Major advances in non-invasive imaging enable the investigation of new aspects of the microcirculation and with ^82^Rb cardiac PET/CT it is possible to measure myocardial flow reserve, reflecting both the function of the large epicardial arteries and the microcirculation. We report an association between cardiac adipose tissue and myocardial flow reserve in non-diabetic controls, but not in persons with type 1 or type 2 diabetes. The few published reports on myocardial flow reserve and cardiac adipose tissue are in line with our results with divergent findings in non-diabetes and diabetes cohorts. A retrospective study, including 46 persons with and 153 persons without diabetes all evaluated for coronary artery disease, observed an association between myocardial flow reserve and epicardial adipose tissue only in the group of persons without diabetes [12]. In a mixed population of 85 persons (28 with diabetes) referred for ^82^Rb PET/CT imaging for clinical indications, retrospective analysis revealed an association between higher epicardial adipose tissue and presence of impaired myocardial flow reserve (myocardial flow reserve ≤ 2) in the total cohort [11].

Why do we find associations between cardiac adipose tissue and CAC and myocardial flow reserve in the controls and not in the persons with diabetes? We speculate that (1) pathophysiological processes promoting coronary atherosclerosis and microvascular dysfunction other than paracrine effects of cardiac adipose tissue are dominant in diabetes; or (2) medical treatment may counteract the adverse effects of the cardiac adipose tissue. Differences in medication between controls and persons with diabetes could then explain the divergent findings.

We report no relationship between cardiac adipose tissue and cardiac sympathetic nerve integrity. To the best of our knowledge, this has not been investigated in persons with diabetes before.

How might our findings impact on clinical practice? Our findings indicate that total cardiac adipose tissue is not a parameter for use in the clinical setting as a marker of microvascular (myocardial flow reserve) or macrovascular (CAC) cardiovascular disease in persons with diabetes.

### Cardiac adipose tissue as treatment target

As the epicardial adipose tissue is a transducer of the harmful effects of systemic inflammation and metabolic disorders on the heart it may represent an important treatment target [10, 23]. A recent study analyzed the adipose tissue surrounding coronary arteries during coronary artery bypass grafting interventions; Two important findings were demonstrated (1) there was increased inflammation of the adipose tissue surrounding the coronary arteries during acute myocardial infarction; and (2) treatment with metformin had an ameliorative effect on the inflammation in the peri-coronary fat and reduced the risk of major cardiovascular events at 12-month follow-up in persons with prediabetes and acute myocardial infarction [24]. Along these lines, a recent study performed in a clinical setting during coronary angiography in non-obstructive coronary stenosis, demonstrated favourable effects of metformin on coronary endothelial dysfunction as well as cardiovascular event-rate in persons with prediabetes [25].

### Study limitations

We applied robust methodology: CT acquisition and assessment followed standard methods and cardiac adipose tissue was automatically quantified using the cardiac software, Syngo.via Frontier [26, 27]. The volumes of cardiac adipose tissue observed in this study were consistent with those previously reported [6, 28–30]. Standardised and validated methodology was used to assess myocardial flow reserve and CAC score. Cardiac ^123^I-MIBG scintigraphy was used for direct assessment of cardiac sympathetic integrity. However, we acknowledge that our study has limitations. The limited size of our study population was only powered to detect stronger correlations and accordingly we may have overlooked smaller correlations. Nevertheless, as the control population was smaller than the diabetic populations an even lower power was present for the controls where we did find the anticipated associations. Also, our sample size is large compared to clinical studies applying similar advanced imaging modalities in persons with diabetes [11, 12]. Estimates of cardiac adipose tissue are known to be subject to inter and intra observer variability. We applied validated methods and automatic analysis to ensure consistency of the volumes measured within our cohort. Nevertheless, we did not differentiate between epi- and pericardial fat, which is a limitation as previous studies found different associations between measures of systolic and diastolic function with epicardial and pericardial adipose tissue [28]. Finally, the only information on systolic function was left ventricle ejection fraction.

## Conclusion

In contrast to what was found in healthy controls, we could not establish a relation between cardiac adipose tissue and coronary calcification or myocardial microvascular function in person with type 1 or type 2 diabetes. The role of cardiac adipose tissue in cardiovascular disease in diabetes remains unclear.

## Data Availability

Data for the present analysis can be provided by the first author on reasonable request.

## Abbreviations

CAC: Coronary artery calcium
CT: Computed tomography
eGFR: Estimated glomerular filtration rate
IQR: Interquartile range
MIBG: Metaiodobenzylguanidine
PET: Positron emission tomography
UACR: Urinary albumin creatinine rate
UAER: Urinary albumin excretion rate
